# Supplementary material for: Sex and age differences in cardiovascular risk factors and lifestyle in patients recently diagnosed with diabetes mellitus: A cross-sectional study in Spanish primary health care
Source: PLoS One. 2025 Feb 13;20(2):e0314519. doi: 10.1371/journal.pone.0314519 (PMC11825014; doi:10.1371/journal.pone.0314519)
Supplement: S2 Table — (DOCX) [file pone.0314519.s002.docx]

**S2 Table. Lifestyle characteristics by age group and sex.**

| Lifestyle characteristics | ≤60 years  N=276 | | | >60 years  N=405 | |  |
| --- | --- | --- | --- | --- | --- | --- |
|  | **Men**  **N=174**  **(63%)** | **Women**  **N=102**  **(37%)** | **p value** | **Men**  **N=208**  **(51.4%)** | **Women**  **N=197**  **(48.6%)** | **p value** |
| Physical activity, n (%) |  |  |  |  |  |  |
| Low | 92 (52.9) | 42 (41.2) | 0.060 | 83 (39.9) | 115 (58.4) | **<.001** |
| Moderate | 60 (34.5) | 44 (43.1) | 0.157 | 100 (48.1) | 72 (36.5) | **0.018** |
| High | 10 (5.7) | 8 (7.8) | 0.5021 | 11 (5.3) | 2 (1.0) | **0.013** |
| Unknown | 12 (6.9) | 8 (7.8) | 0.782 | 14 (6.9) | 8 (4.1) | 0.217 |
| Smoking, n/N’ (%) |  |  |  |  |  |  |
| Active smoker | 50 (28.7) | 11 (10.8) | **<.001** | 37 (17.8) | 25 (12.7) | 0.154 |
| Ex smoker | 48 (27.6) | 29 (28.4) | 0.886 | 113 (54.3) | 60 (30.5) | **<.001** |
| Nonsmoker | 63 (36.2) | 54 (52.9) | **<.001** | 45 (21.6) | 105 (53.3) | **<.001** |
| Unknown | 12 (7.5) | 8 (7.8) | 0.928 | 13 (6.3) | 7 (3.6) | 0.211 |
| Alcohol, n/N’ (%) |  |  |  |  |  |  |
| Heavy drinker | 11 (6.3) | 0 (0.0) | **0.004** | 16 (7.7) | 3 (1.5) | **<.01** |
| Drinker, but not risky | 70 (40.2) | 26 (25.5) | **0.012** | 114 (54.8) | 60 (30.5) | **<.001** |
| Teetotal | 79 (45.4) | 68 (66.7) | **<.001** | 60 (28.8) | 122 (61.9) | **<.001** |
| Unknown | 14 (8.0) | 8 (7.8) | 0.953 | 18 (8.7) | 12 (6.1) | 0.318 |
| Alcohol units per week, median (interquartile range) | 0.4 (0.0-5.8) | 0.0 (0.0-75.0) | **<.001** | 2.0 (0.0-10.1) | 0.0 (0.0-0.7) | **<.001** |
| MEDAS Score *◊*, n/N’ (%) |  |  |  |  |  |  |
| Low adherence, 0-5 | 19/159 (11.9) | 3/93 (3.2) | **0.012** | 12/193 (6.2) | 5/186 (2.7) | 0.099 |
| Medium adherence, 6-10 | 109/159 (68.6) | 64/93 (68.8) | 0.974 | 131/193 (67.9) | 136/186 (73.1) | 0.267 |
| High adherence ≥11 | 31/159 (19.5) | 26/93 (28.0) | 0.126 | 50/193 (25.9) | 45/186 (24.2) | 0.703 |
| 14-MEDAS Questionnaire, n/N’ (%) |  |  |  |  |  |  |
| Olive oil as main source of fat | 147/161 (91.3) | 86/93 (92.5) | 0.745 | 190/194 (97.9) | 184/189 (97.4) | 0.706 |
| Consumption of more than 3 tablespoons of olive oil per day | 80/161 (49.7) | 42/93 (45.2) | 0.487 | 94/194 (48.5) | 96/188 (51.1) | 0.610 |
| Consumption of vegetables per day,  more than 1 portion | 75/161 (46.6) | 60/93 (64.5) | **<.01** | 97/194 (50.0) | 115/188 (61.2) | **0.028** |
| Consumption of fruits per day,  more than 2 | 75/161 (46.6) | 62/93 (66.7) | **<.01** | 116/193 (60.1) | 139/188 (73.9) | **<.01** |
| Consumption of red meat per day,  less than 1 | 102/160 (63.8) | 72/93 (77.4) | **0.024** | 147/194 (75.8) | 139/187 (74.3) | 0.745 |
| Consumption butter per day,  less than 1 | 115/160 (71.9) | 70/93 (75.3) | 0.557 | 139/194 (71.6) | 141/188 (75.0) | 0.459 |
| Consumption sugary drinks per day,  less than 1 | 105/161 (65.2) | 68/93 (73.1) | 0.193 | 132/194 (68.0) | 132/188 (70.2) | 0.646 |
| Consumption wine per week,  more than 2 glasses | 22/160 (13.8) | 3/93 (3.2) | **<.01** | 61/194 (12.4) | 12/188 (6.4) | **<.001** |
| Consumption legumes per week,  more than 2 portions | 95/160 (59.4) | 50/93 (53.8) | 0.384 | 119/194 (61.3) | 105/188 (55.9) | 0.276 |
| Consumption fish per week,  more than 2 | 92/160 (57.5) | 51/93 (54.8) | 0.681 | 115/194 (59.3) | 123/187 (65.8) | 0.190 |
| Consumption sweets per week,  less than 3 | 110/159 (69.2) | 73/93  (78.5) | 0.110 | 129/194 (66.5) | 137/188 (72.9) | 0.175 |
| Consumption nuts per week, at least 1 | 98/160 (61.3) | 53/93 (57.0) | 0.505 | 109/194 (56.2) | 87/188 (46.3) | 0.053 |
| Consumption preferably white meat per week | 123/160 (76.9) | 86/93  (92.5) | **<.01** | 142/194 (73.2) | 165/188 (87.8) | **<.01** |
| Consumption sautéed vegetables per week, more than once | 101/160 (63.1) | 56/93  (60.2) | 0.646 | 123/194 (63.4) | 123/188 (65.4) | 0.680 |

*Comparisons between groups were performed using the χ2 test for categorical variables and the t test for continuous variables. The Z test was used for comparisons between subcategories. Cardiovascular disease considers coronary heart disease, peripheral artery disease and cerebrovascular disease. n/N’: number of cases divided by total number of participants with available information. Bold values denote statistical significance.* ** The measure of variables that do not conform to a normal distribution is given in median (interquartile range) and comparison were performed using Mann-Whitney U test. ◊ MEDAS Score categories: Low adherence=0-5 points; Medium adherence=6-10; High adherence=11-15.*
